# Rapid and Accurate Antibiotic Susceptibility Determination of *tet*(X)-Positive E. coli Using RNA Biomarkers

**DOI:** 10.1128/Spectrum.00648-21

**Published:** 2021-10-27

**Authors:** Haijie Zhang, Yan Li, Yongjia Jiang, Xiaoyu Lu, Ruichao Li, Daxin Peng, Zhiqiang Wang, Yuan Liu

**Affiliations:** a College of Veterinary Medicine, Yangzhou University, Yangzhou, China; b Jiangsu Co-innovation Center for Prevention and Control of Important Animal Infectious Diseases and Zoonoses, Yangzhou University, Yangzhou, China; c Institute of Comparative Medicine, Yangzhou University, Yangzhou, China; d Joint International Research Laboratory of Agriculture and Agri-Product Safety, the Ministry of Education of China, Yangzhou University, Yangzhou, China; Montefiore Medical Center and Albert Einstein College of Medicine

**Keywords:** antibiotic resistance, *tet*(X), tigecycline, antibiotic susceptibility determination, bacteria

## Abstract

The emergence and prevalence of novel plasmid-mediated tigecycline resistance genes, namely, *tet*(X) and their variants, pose a serious threat to public health worldwide. Rapid and accurate antibiotic susceptibility testing (AST) that can simultaneously detect the genotype and phenotype of *tet*(X)-positive bacteria may contribute to the deployment of an effective antibiotic arsenal, mortality reduction, and a decrease in the use of broad-spectrum antimicrobial agents. However, current bacterial growth-based AST methods, such as broth microdilution, are time consuming and delay the prompt treatment of infectious diseases. Here, we developed a rapid RNA-based AST (RBAST) assay to effectively distinguish *tet*(X)-positive and -negative strains. RBAST works by detecting specific mRNA expression signatures in bacteria after short-term tigecycline exposure. As a proof of concept, a panel of clinical isolates was characterized successfully by using the RBAST method, with a 3-h assay time and 87.9% accuracy (95% confidence interval [CI], 71.8% to 96.6%). Altogether, our findings suggest that RNA signatures upon antibiotic exposure are promising biomarkers for the development of rapid AST, which could inform early antibiotic choices.

**IMPORTANCE** Infections caused by multidrug-resistant (MDR) Gram-negative pathogens are an increasing threat to global health. Tigecycline is one of the last-resort antibiotics for the treatment of these complicated infections; however, the emergence of plasmid-encoded tigecycline resistance genes, namely, *tet*(X), severely diminishes its clinical efficacy. Currently, there is a lack of rapid and accurate antibiotic susceptibility testing (AST) for the detection of *tet*(X)-positive bacteria. In this study, we developed a rapid and robust RNA-based antibiotic susceptibility determination (RBAST) assay to effectively distinguish *tet*(X)-negative and -positive strains using specific RNA biomarkers in bacteria after tigecycline exposure. Using this RBAST method, we successfully characterized a set of clinical strains in 3 h. Our data indicate that the RBAST assay is useful for identifying *tet*(X)-positive Escherichia coli.

## INTRODUCTION

The spread of antibiotic resistance has been an increasing global concern that seriously threatens human health and biosecurity in the 21st century (1, 2). Tigecycline (3), a semisynthetic parenteral glycylcycline, is considered one of the last options for the treatment of severe infections caused by multidrug-resistant (MDR) bacterial pathogens, particularly carbapenem-resistant *Enterobacteriaceae* (CRE) (4) and MCR-producing pathogens (5). However, the clinical efficacy of this last-resort antibiotic has been challenged by the appearance of newly identified mobile tigecycline resistance genes called *tet*(X3/X4), which confer high-level tigecycline resistance in various Gram-negative microorganisms (6, 7).

Tet(X) and its variants are resistance enzymes that perform a unique tetracycline modification mechanism (8). Specifically, *tet*(X) encodes a flavin-dependent monooxygenase that can selectively hydroxylate the tigecycline substrate at C11a (9), resulting in the production of 11a-hydroxy tigecycline and thereby inactivating all tetracyclines, including newly FDA-approved eravacycline and omadacycline (6, 7, 10). Since the first report of mobile *tet*(X3) and *tet*(X4) genes in *Enterobacteriaceae* and Acinetobacter isolates from China in 2019, an alarming number of strains carrying *tet*(X3/X4) resistance determinants have been isolated from animals and meat and even from secretion samples of inpatients (7, 11). To date, *tet*(X3/X4) genes have been found in eight bacterial species isolated from animals, humans, food, and the environment (7, 12). More alarmingly, most plasmid-mediate *tet*(X4)-positive Escherichia coli are MDR bacteria and are reportedly resistant to three or more different classes of antimicrobials (13, 14). Simultaneously, some of them are confirmed to be resistant to colistin (11, 15). Moreover, we previously identified an animal-origin Proteus cibarius strain coharboring a *tet*(X6) variant and a *bla*_NDM-1_ gene (16). The transmission of *tet*(X) and its variants could potentially lead to an increasing risk of infection and antibiotic treatment failure in humans and animals (13). Because we are facing these challenges, fast and accurate antibiotic susceptibility testing (AST) is urgently needed to identify tigecycline-resistant pathogens mediated by *tet*(X) genes.

Traditional culture-based methods for AST are highly dependent on the arrest of the growth of living cells in the presence of different concentrations of antibiotics. These methods include the agar dilution or the minimal broth dilution method, which are time consuming (requires an 18-hour incubation) and necessitate that bacterial cells be quantified accurately (17). Recent progress in diagnostic technologies, including nucleic acid amplification assays, microfluidics, and biosensors, are pushing timescale boundaries for diagnosing bacterial infections (18). Moreover, next-generation sequencing technology has been improving the scale of known resistance genes; however, it cannot provide information on the antibiotic resistance phenotype (19). Unlike DNA, the RNA transcriptome has the potential to provide accelerated phenotypic susceptibility information independent of cell division. Transcriptional responses are one of the earliest cellular changes during antibiotics exposure and occur far before observable phenotypic changes in growth (20). Accordingly, the RNA transcriptome and specific RNA biomarkers have been used for the identification of antibiotic susceptibility and resistance in Klebsiella pneumoniae and Acinetobacter baumannii after antibiotic exposure (21, 22). Quantifying changes in RNA biomarkers is hence a particularly appealing approach for the development of rapid and accurate AST.

In this study, we successfully established a rapid and accurate RNA-based AST (RBAST) assay to detect both *tet*(X)-positive and tigecycline-resistant bacteria using a bacterium-antibiotic model. This rapid AST method is developed based on significantly different transcriptome responses of an engineered tigecycline-susceptible strain (DH5α-pUC19) and *tet*(X4)-mediated tigecycline-resistant strain [DH5α-pUC19-*tet*(X4)] after a 60-min tigecycline exposure. The candidate RNA biomarkers used in RBAST were verified using reverse-transcription quantitative PCR (qRT-PCR) across temporal and tigecycline concentration shifts. Further validations of the selected RNA biomarkers in other variants of *tet*(X) and clinical isolates were also performed upon tigecycline exposure. As a proof of concept, a panel of clinical isolates was characterized by using the RBAST method, and the results were compared with traditional MIC determination and PCR analysis.

## RESULTS

### *tet*(X4)-positive and -negative bacteria display different transcriptome profiles.

**FIG 1:**
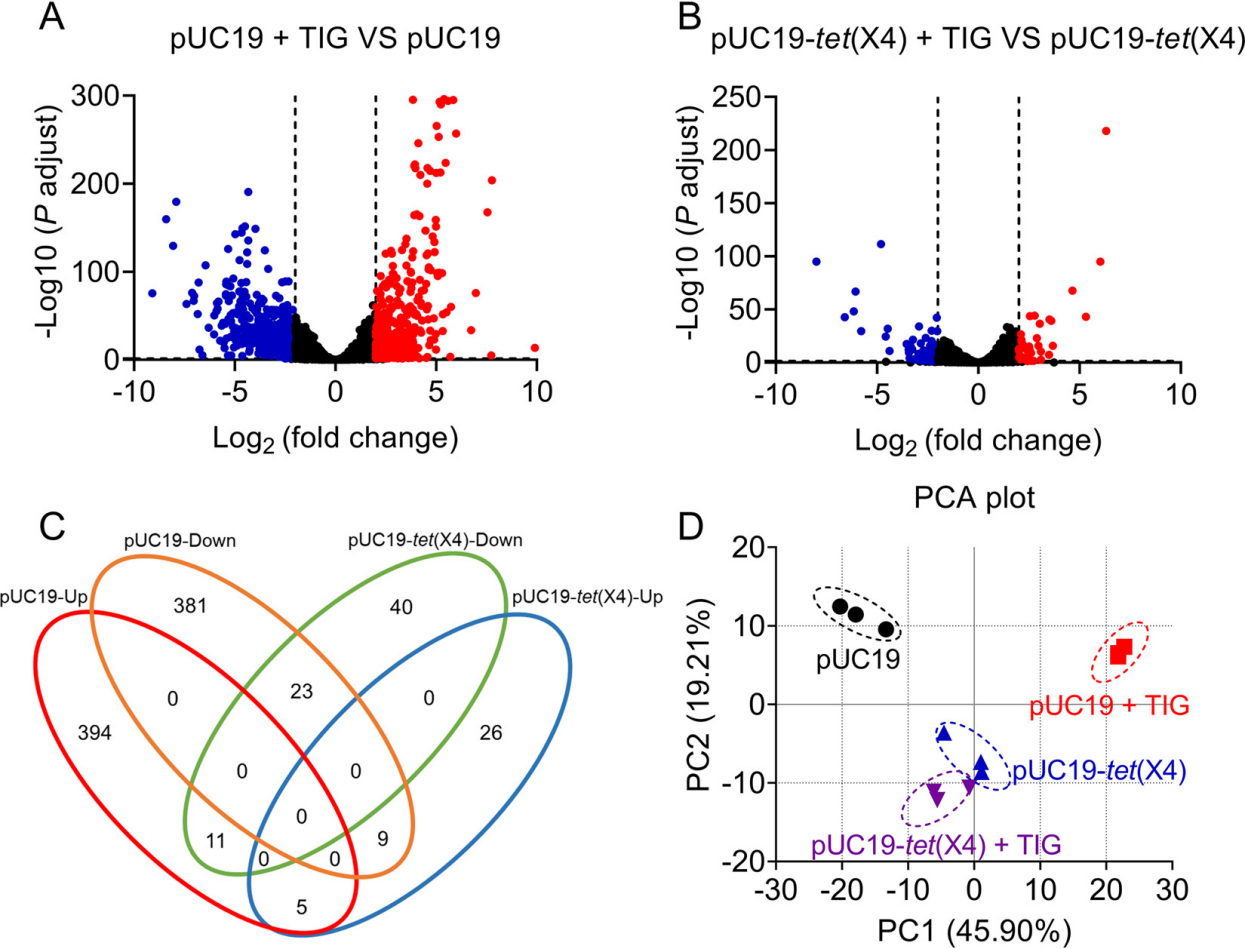
Differential gene expression of *tet*(X4)-negative and -positive strains upon antibiotic exposure. (A and B) Scatter diagram of differentially expressed genes from *tet*(X4)-negative tigecycline-susceptible (DH5α-pUC19) and *tet*(X4)-positive tigecycline-resistant [DH5α-pUC19-*tet*(X4)] strains relative to their own controls. Red points indicate upregulated genes, and blue points indicate downregulated genes. (C) Venn diagrams display the number of metabolites significantly affected by *tet*(X4)-negative (DH5α-pUC19) and *tet*(X4)-positive [DH5α-pUC19-*tet*(X4)] strains after tigecycline exposure relative to their own control. FDR, <0.05; log_2_FC, ≤−2 or ≥ 2; *P < *0.05 (one-way ANOVA). (D) PCA score plots of the first four principal components for metabolite levels from *tet*(X4)-negative (DH5α-pUC19) and *tet*(X4)-positive [DH5α-pUC19-*tet*(X4)] strains with or without tigecycline exposure.

To identify the specific transcripts that effectively distinguish *tet*(X4)-positive and -negative bacteria, transcriptome profiling of the engineered *tet*(X)-negative strain (DH5α-pUC19) and *tet*(X4)-mediated tigecycline-resistant strain [DH5α-pUC19-*tet*(X4)] was performed. Because the doubling time of E. coli is approximately 20 to 60 min (23), the tigecycline treatment was set at 60 min to make sure that the exposure procedure was long enough for E. coli to make significant changes in the transcriptome profile. As shown in Fig. 1A and B, a series of significantly altered transcripts can be observed after the 60-min tigecycline exposure (2 μg/ml). Global shifts in RNA expression were also observed in the DH5α-pUC19 after tigecycline exposure, including 410 upregulated and 413 downregulated genes (Fig. 1A). However, only 40 upregulated and 74 downregulated genes were identified in the DH5α-pUC19-*tet*(X4) group (Fig. 1B), indicating that *tet*(X4)-positive bacteria had a low-level response to tigecycline exposure compared with *tet*(X4)-negative microorganisms. The genes with significantly altered expression (false discovery rate [FDR], <0.05; log_2_ fold change [FC], ≥2 or ≤−2; *P < *0.05, analysis of variance [ANOVA]) were sorted out for further analysis. In detail, only 16 and 32 upregulated and downregulated genes, respectively, were altered in the pUC19-*tet*(X4) group (Fig. 1C). To further illustrate whether tigecycline could trigger distinct gene expression profiles between the DH5α-pUC19 and DH5α-pUC19-*tet*(X4) groups, a principal-component analysis (PCA) was performed using gene lists from control and treated samples. The PCA results demonstrated that a considerably diverse gene expression profile was induced in the control and treated DH5α-pUC19 groups, whereas a less diverse gene expression profile was observed in the DH5α-pUC19-*tet*(X4) group (Fig. 1D). These results suggest that *tet*(X4)-positive and -negative bacteria exhibit different shifts in global gene lists after tigecycline exposure at the breakpoint concentration.

### Functional enrichment of differentially expressed genes.

**FIG 2:**
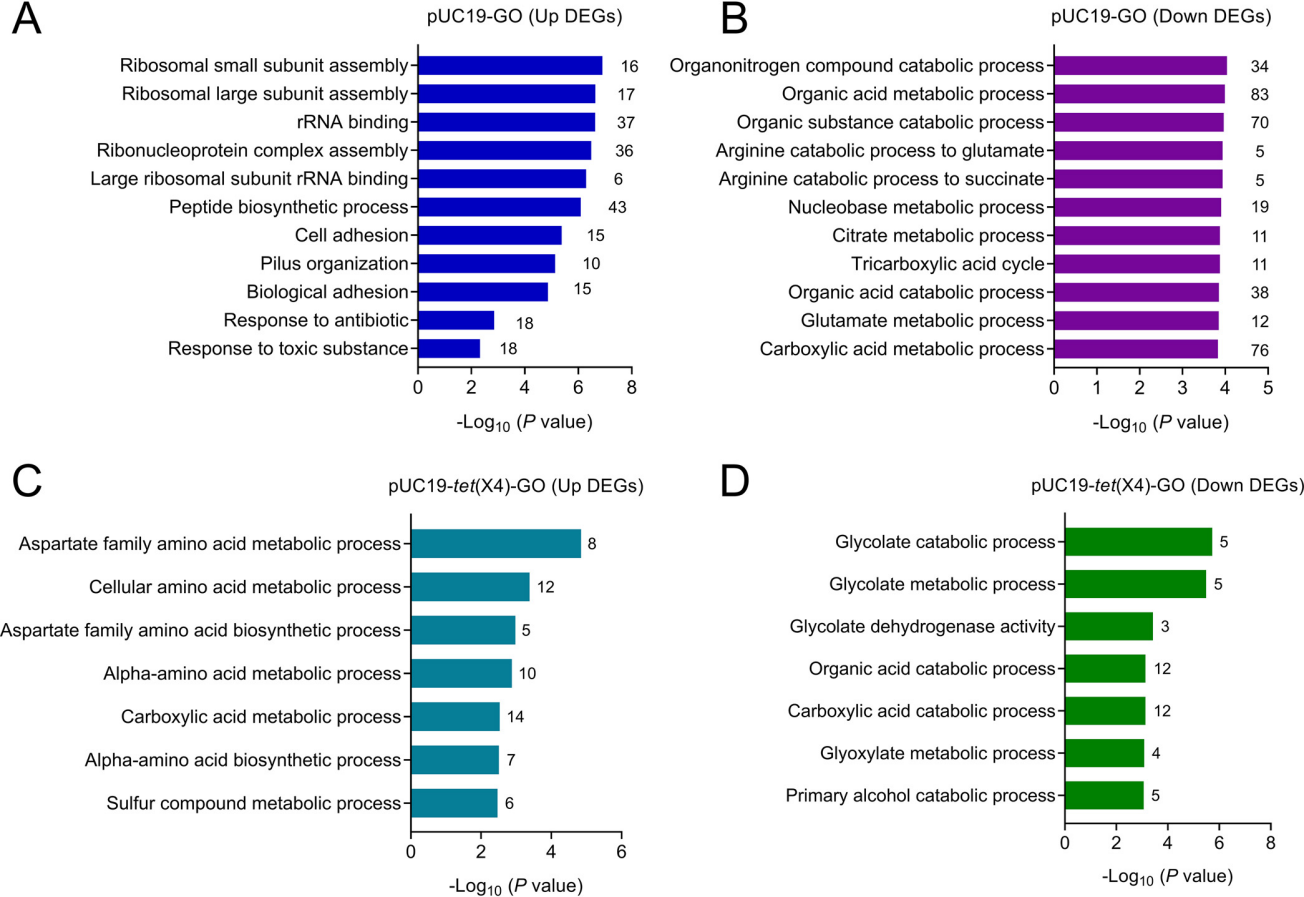
Functional enrichment of differentially expressed genes upon antibiotic exposure. GO pathway enrichment in *tet*(X4)-negative tigecycline-susceptible (DH5α-pUC19) (A and B) and *tet*(X4)-positive tigecycline-resistant [DH5α-pUC19-*tet*(X4)] (C and D) groups after tigecycline exposure relative to their own control. FDR, <0.05; log_2_FC, ≤−2 or ≥2; *P < *0.05 (one-way ANOVA).

To better understand the functional enrichment of the transcriptome profile, general Gene Ontology (GO) analyses were retrieved for functional annotation (Fig. 2). The results revealed that tigecycline-specific susceptible genes were enriched in the DH5α-pUC19 group in response to antibiotic and toxic substances, demonstrating the initiation of cell death after tigecycline (2 μg/ml) exposure, however, this result was not observed in the *tet*(X4)-positive group [DH5α-pUC19-*tet*(X4)]. Tigecycline acts by reversibly binding to the ribosome 30S subunit and inhibits the translation elongation step by preventing amino acylated tRNAs to accommodate in the ribosomal A site. Consistent with the modes of action of tigecycline, the ribosomal small and large subunit assembly, rRNA binding, cellular macromolecular complex assembly, and large ribosomal subunit rRNA binding-related pathways were highly upregulated in the tigecycline-treated *tet*(X4)-negative group but not in the *tet*(X4)-mediated tigecycline-resistant group. Furthermore, global transcriptional data showed consistent tigecycline-induced downregulation of gene expression for enzymes in the tricarboxylic acid (TCA) cycle. The transcription of genes responsible for the catabolism of organic substances, including amino acids, organonitrogen, nucleobase, and glutamate, as well as for the TCA cycle and other metabolic pathways, such as cellular respiration, were also downregulated by tigecycline in the DH5α-pUC19 group (Fig. 2A and B) but not in the DH5α-pUC19-*tet*(X4) group (Fig. 2C and D). These data confirm that the *tet*(X4)-negative microorganisms (DH5α-pUC19) exhibit a significant cell arrest under tigecycline exposure, whereas *tet*(X4)-positive bacteria [DH5α-pUC19-*tet*(X4)] have a minimum response in the presence of tigecycline at the breakpoint concentration.

### Selection of candidate RNA biomarkers for rapid AST.

**FIG 3:**
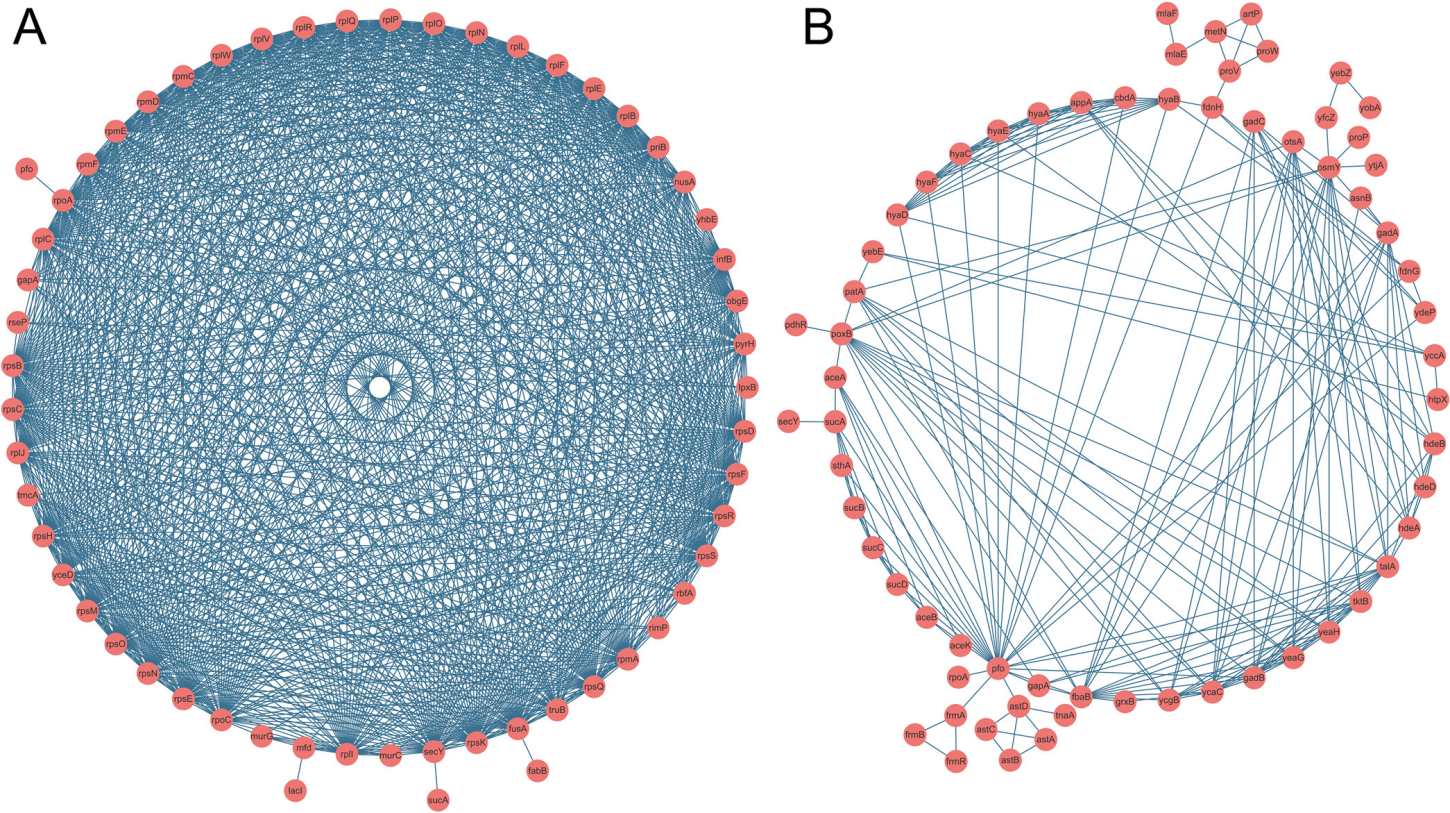
Two significantly enriched networks of tigecycline-specific susceptible genes were identified using STRING-db. (A) Tigecycline module 1 terms about ribosomal or ribonucleoprotein assembly. (B) Tigecycline module 2 terms, including tricarboxylic acid cycle, citrate metabolic process, and arginine catabolic process.

Considering the heterogeneous RNA transcripts between the DH5α-pUC19 and DH5α-pUC19-*tet*(X4) groups in response to tigecycline treatment, we hypothesized whether these differentially expressed genes could serve as specific RNA biomarkers for a rapid molecular AST for *tet*(X4)-positive strains. To test this hypothesis, the candidate tigecycline-specific susceptible gene list was generated by including genes with significant differential expression levels compared with the control DH5α-pUC19 group (see Table S3 in the supplemental material). A minimum log_2_FC of ≤−2 or ≥2 was required in the transcriptome profiles, which were sorted by the *P* value (*P < *0.05). To better understand the biological function of these tigecycline-specific susceptible genes, a network analysis was performed by grouping them into coexpression modules using STRING-db v.10.5 (https://string-db.org/). Two coexpression modules were identified for these 122 tigecycline-specific susceptible genes after tigecycline exposure (Fig. 3). A total of 57 genes concentrated in module 1 (Fig. 3A) were enriched in the upregulated groups with respect to the pUC19-GO analysis terms about ribosomal or ribonucleoprotein assembly. In contrast, 65 genes in module 2 (Fig. 3B) were enriched in the downregulated terms, including the TCA cycle, citrate metabolic process, and arginine catabolic process. These results indicate that the tigecycline-specific susceptible genes are associated primarily with the elongation step of protein translation and bacterial metabolisms.

### Validation of candidate RNA biomarkers in E. coli clinical isolates.

**FIG 4:**
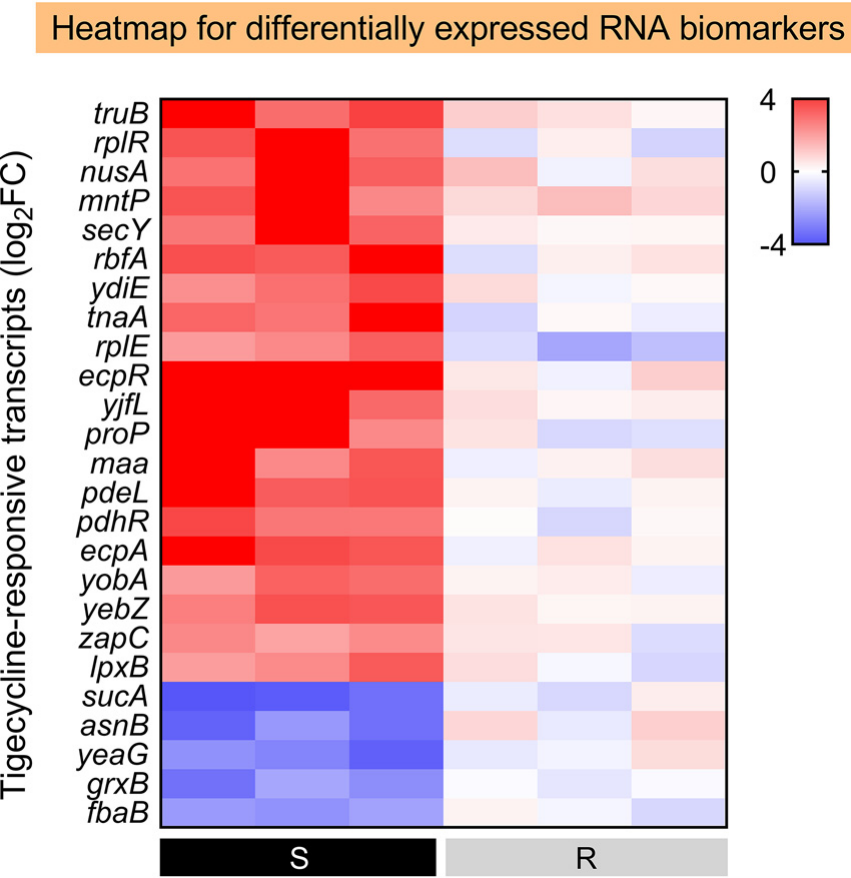
RBAST distinguishes *tet*(X4)-negative and -positive clinical strains. Heatmap of 25 tigecycline-sensitive RNA biomarkers across three *tet*(X4)-negative and three *tet*(X4)-positive tigecycline-resistant E. coli after tigecycline exposure relative to their own control. Left three black panels indicate *tet*(X4)-negative strains, and right three gray panels indicate *tet*(X4)-positive isolates. 16S rRNA was used as a reference gene.

Three *tet*(X4)-negative strains and three *tet*(X4)-positive tigecycline-resistant E. coli isolates with a clear genetic background were used for qRT-PCR-based verification to confirm whether the chosen candidate RNA biomarkers are applicable for a rapid molecular AST. A minimum log_2_FC of ≤−2 or ≥2 was required for biomarkers to be “significantly differential” in the quantitative analysis using qRT-PCR. The primers are shown in Table S4 in the supplemental material. According to Fig. 4 and Fig. S1 in the supplemental material, 25 out of 139 candidate RNA biomarkers showed significant differential expression levels between the *tet*(X4)-negative and -positive groups after tigecycline treatment. In particular, 20 (*truB*, *yfcC*, *marR*, *mntP*, *suhB*, *lpxB*, *ybjG*, *ydiE*, *tnaA*, *rplE*, *ecpR*, *yjfL*, *prop*, *maa*, *pdeL*, *pdhR*, *ecpA*, *yobA*, *yebZ*, and *grxB*) out of 25 selected genes were identified as significantly upregulated RNA biomarkers in all 3 chosen *tet*(X4)-negative strains, whereas all 3 *tet*(X4)-positive isolates had no significant changes. In addition, only five genes (*sucA*, *asnB*, *yeaG*, *fbaB*, and *zapC*) were identified as significantly downregulated RNA biomarkers in all three *tet*(X4)-negative strains, but none were identified in all three *tet*(X4)-positive tigecycline-resistant isolates.

### Tigecycline concentration shifts in selected RNA biomarkers upon tigecycline exposure.

**FIG 5:**
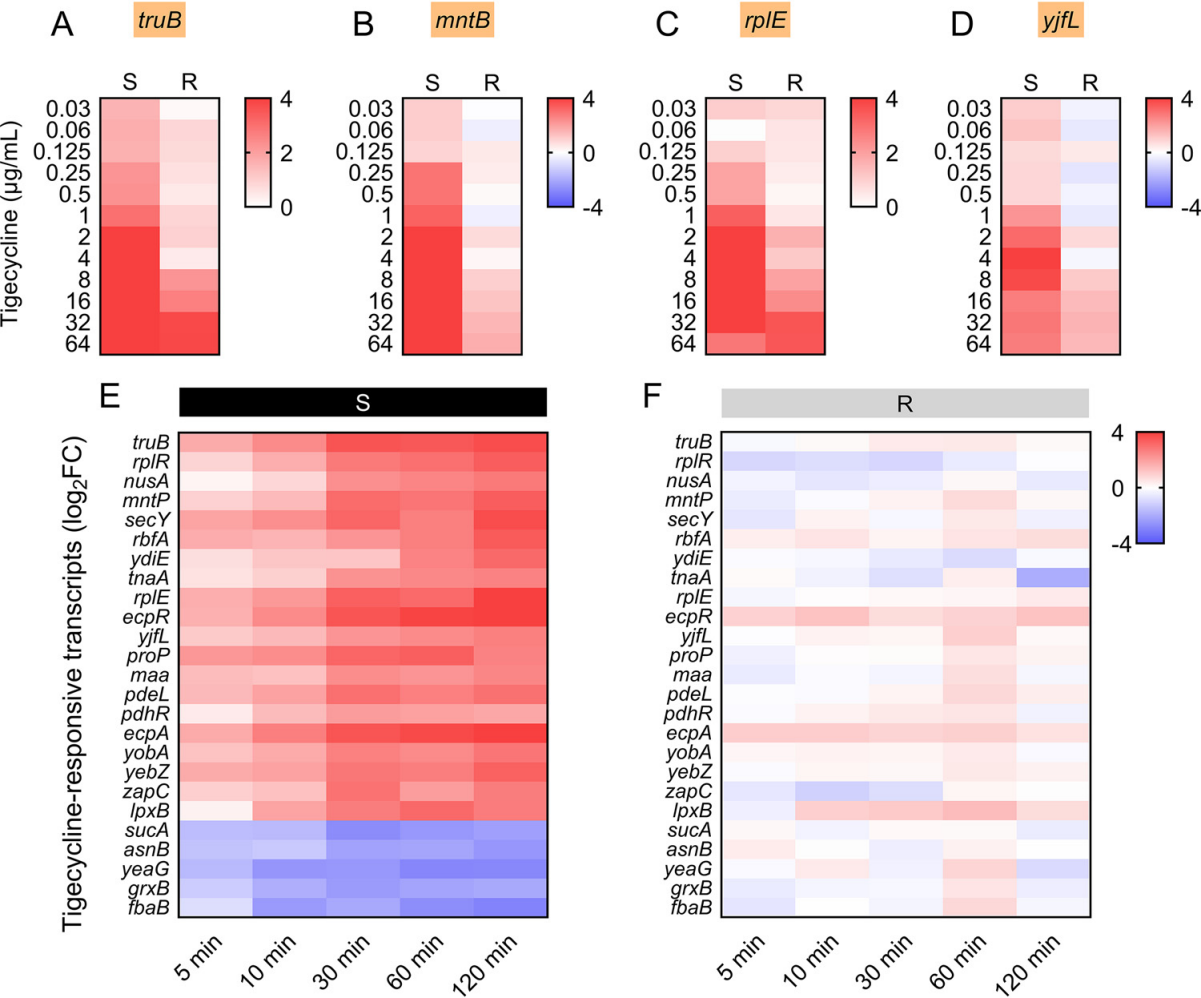
Expression of selected RNA biomarkers upon different antibiotic exposure concentrations and times. Heatmap of *truB* (A), *mntB* (B), *rplE* (C), and *yjfL* (D) biomarkers demonstrated the most sensitive information across the MIC range of tigecycline. Heatmap of 25 differentially expressed RNA biomarkers across exposure duration of tigecycline (E and F). Left black panels indicate *tet*(X4)-negative E. coli TACC25922, and right gray panels indicate *tet*(X4)-positive isolate RW7-1. 16S rRNA was used as a reference gene.

Having shown that 25 candidate RNA biomarkers in *tet*(X4)-negative and -positive bacteria displayed completely different responses to tigecycline treatment at the breakpoint concentration (2 μg/ml), we subsequently investigated whether the tigecycline concentration shifts would affect the potential of these RNA signatures as detection biomarkers. To this end, a standard susceptible E. coli ATCC 25922 and a validated *tet*(X4)-positive E. coli isolate were exposed to a wide range of tigecycline concentrations (ranging from 0.03125 to 64 μg/ml). Total RNA was extracted after a 60-min exposure, followed by qRT-PCR of the selected RNA biomarkers. Specifically, *truB*, *mntB*, *rplE*, and *yjfL* exhibited increasing regulation in a dose-dependent manner as long as the tigecycline concentrations were high enough (Fig. 5A to D; see Fig. S2A to D in the supplemental material). The MICs of the *tet*(X4)-negative E. coli isolates ranged from 0.25 to 0.5 μg/ml. When the concentration of tigecycline exceeded 0.25 μg/ml, the RNA biomarkers were identified as significantly upregulated in the chosen *tet*(X4)-negative isolates, whereas no change was observed in the *tet*(X4)-positive bacteria. Consistently, the RNA biomarkers in the *tet*(X4)-positive isolates responded similarly to those in the tigecycline-susceptible isolates, as the tigecycline concentrations went up to 8 to 16 μg/ml, which correspond to the MICs of tigecycline-resistant E. coli isolates. These results further support the idea that exposure to tigecycline at the breakpoint concentration is a feasible means of screening specific mRNA expression signatures between *tet*(X4)-negative and -positive bacteria.

### Temporal shifts in selected RNA biomarkers upon tigecycline exposure.

Next, we evaluated the effect of incubation time on the expression of selected RNA biomarkers. A standard susceptible E. coli ATCC 25922 isolate and a validated *tet*(X4)-mediated tigecycline-resistant E. coli isolate were exposed to 2-μg/ml tigecycline ranging from 5 to 120 min in the experimental design (23). As shown in Fig. 5E and F and Fig. S3 in the supplemental material, global shifts of 25 selected RNA biomarkers in the *tet*(X4)-negative strain were observed in a very short time (5 min) and reached a peak at about 30 min after tigecycline exposure. However, there was no significant fold change in the *tet*(X4)-positive tigecycline-resistant E. coli isolate after 60 min of stimulation. These data imply that the response of our selected RNA biomarkers to tigecycline treatment is very sensitive and rapid, enabling the detection of *tet*(X4)-positive bacteria in a very short time.

### Validation of selected RNA biomarkers in other variants of *tet*(X).

**FIG 6:**
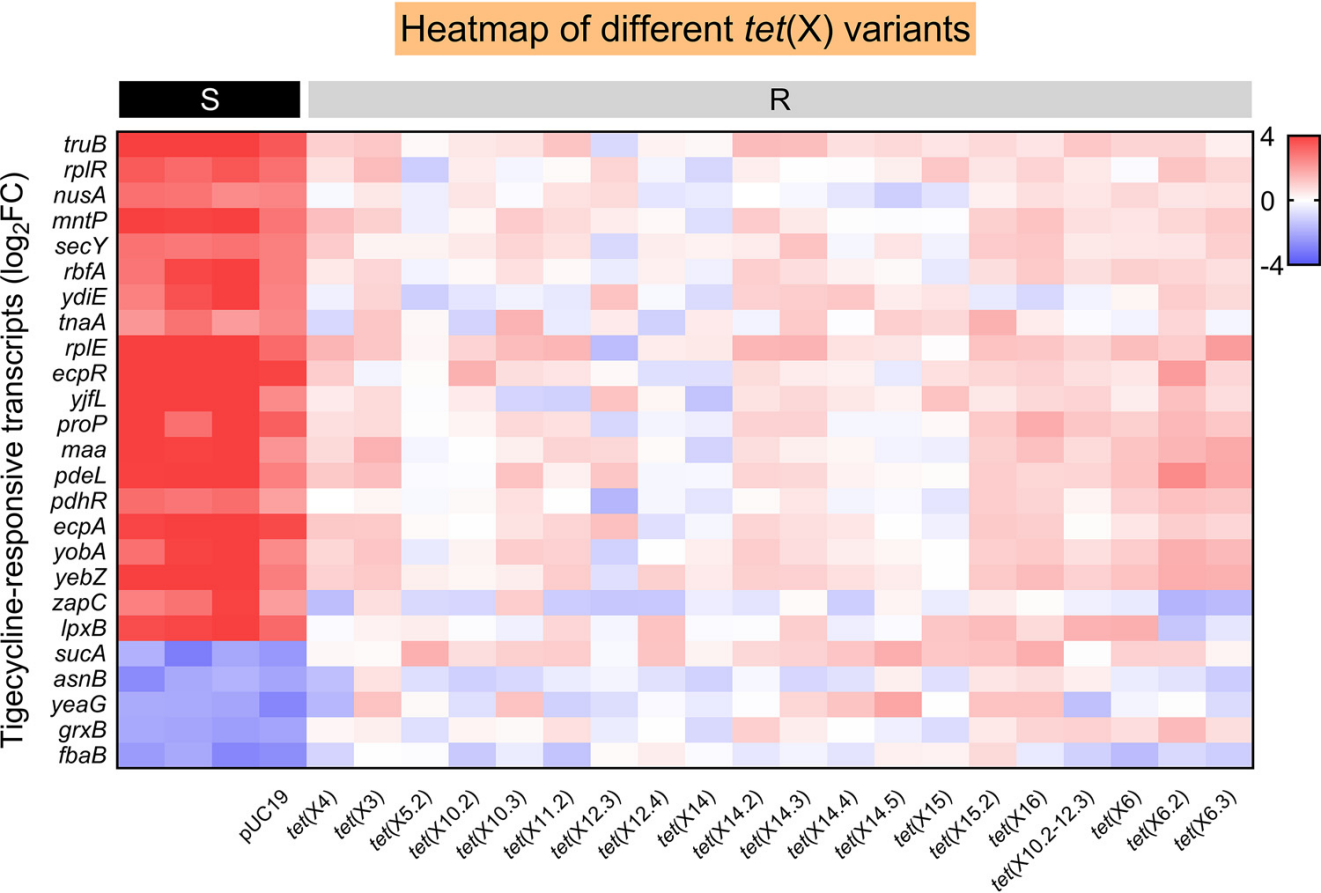
RBAST detects different *tet*(X) variants using the selected RNA biomarkers. Heatmap of 25 differentially expressed RNA biomarkers validated across *tet*(X)-negative strains, and different variants of *tet*(X)-positive tigecycline-resistant strains after tigecycline exposure relative to their own control. Left four black panels indicate *tet*(X)-negative E. coli, and right 20 gray panels indicate the tigecycline-resistant bacteria carrying different *tet*(X) variants. 16S rRNA was used as a reference gene.

To confirm whether the selected RNA biomarkers could be used to identify tigecycline-resistant E. coli isolates caused by other variants of *tet*(X), coding DNA sequences (CDSs) with their promoter of 16 different *tet*(X) variants from the NCBI database were successfully cloned into pUC19 and transformed into DH5α. The CDSs of *tet*(X6), *tet*(X6.2), and *tet*(X6.3) were cloned into pET23 (+) with the T7 promoter and were transformed into E. coli BL21(DE3). The MIC results demonstrated that all constructions displayed an obvious tigecycline resistance phenotype (MIC, ≥8 μg/ml). RNA was extracted after 60 min of tigecycline exposure, followed by qRT-PCR of all 25 selected RNA biomarkers. DH5α with transformed pUC19 was used as a susceptible control. Consequently, a significant differential expression level of selected RNA biomarkers was observed among engineered *tet*(X) variant-positive bacteria after tigecycline treatment. Similar to *tet*(X4)-positive bacteria, all *tet*(X) variant-positive groups had no remarkable response to tigecycline exposure compared with the tigecycline susceptible group (Fig. 6; see Fig. S4 in the supplemental material). Altogether, these results suggest that the selected RNA biomarkers are also applicable for the rapid molecular AST of *tet*(X4) and other *tet*(X) variant-conferred tigecycline-resistant E. coli isolates.

### Accuracy of RBAST in clinical isolates.

**FIG 7:**
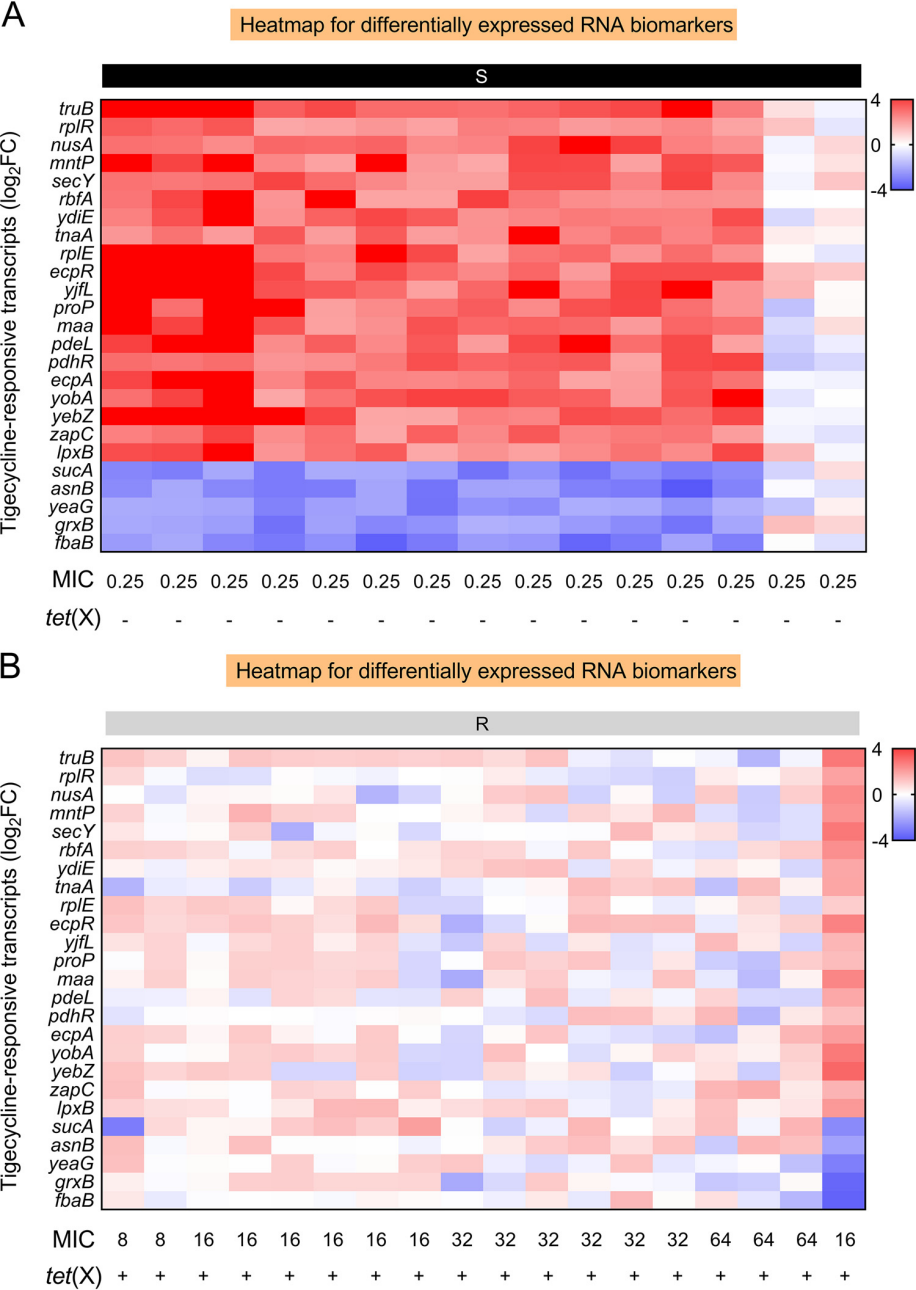
RBAST accurately classifies E. coli isolates. Heatmap of 25 validated RNA biomarkers across clinical *tet*(X)-negative tigecycline-susceptible (A) and *tet*(X)-positive tigecycline-resistant (B) E. coli isolates after tigecycline exposure relative to their own control. 16S rRNA was used as a reference gene.

The experiments mentioned above demonstrate that the selected RNA biomarkers can be used to rapidly distinguish *tet*(X)-mediated tigecycline resistance. To further verify the accuracy and potential application of RBAST in clinical practice, 33 clinical E. coli isolates were selected for RBAST verification, MIC determination, and PCR analysis. For higher accuracy, two features involving a minimum log_2_FC of ≤−2 or ≥2 and upregulation or downregulation of at least 20/25 selected RNA biomarkers in the RBAST analysis were required to define an isolate as *tet*(X) negative. In contrast, isolates without significant differential regulation (−2< log_2_FC <2) in at least 20/25 selected RNA biomarkers were defined as *tet*(X) positive. According to the RBAST results, 14 out of 33 isolates were defined as *tet*(X) negative (Fig. 7A; see Fig. S5 in the supplemental material), whereas 19 were considered *tet*(X) positive (Fig. 7B; see Fig. S6 in the supplemental material). Compared with the MIC determination and PCR analysis, the developed RBAST correctly classified 29 of 33 isolates with 13 of 15 *tet*(X)-negative isolates and 17 of 18 *tet*(X)-positive isolates. The accuracy characterized by the RBAST method during the 3-h assay time was 87.9% (95% confidence interval [CI], 71.8% to 96.6%). Collectively, these data indicate that RBAST can effectively detect *tet*(X)-mediated tigecycline resistance in the clinical setting.

## DISCUSSION

Over the past decades, great efforts have been made to develop diagnostic methods that can rapidly characterize pathogens and their antibiotic susceptibility to meet urgent clinical demand for the treatment of various infections. However, there is still a lack of rapid and accurate methods to address this issue, particularly for the emerging *tet*(X)-positive bacteria. In this study, we first characterized the global transcriptome profile of DH5α-pUC19 and DH5α-pUC19-*tet*(X4) after tigecycline exposure and found that *tet*(X)-negative and -positive strains displayed completely different RNA transcripts. Specifically, antibiotic treatment resulted in drastic transcriptional responses in the *tet*(X)-negative groups within a few minutes, whereas only a few responses were observed in the *tet*(X)-positive groups. Based on these findings, we developed a rapid and accurate AST assay termed RBAST to determine tigecycline susceptibility in bacterial strains using these elicited RNA signatures.

The spread of *tet*(X)-positive bacteria calls for more effective AST methods. Although several typical AST methods have been reported recently for the detection of *tet*(X)-producing E. coli, most of those methods are time consuming and laborious, as well as the separation of genotype and phenotype. These methods are based on bacterial growth, which requires several rounds of enrichment cultivations to increase the number of bacteria. Difficulties in monitoring cell density in suspension and the retarded microbial growth during early stages are time-limiting factors for these traditional AST methods. For example, genotypic detection of *tet*(X) using PCR analysis allows high sensitivity and specificity, but high-throughput detection cannot be achieved owing to the lack of universal primers for each gene subtype; therefore, this method cannot identify unknown genes (24, 25). In addition, Cui et al. developed an approach for detecting *tet*(X)-producing strains after a 3-h incubation of bacterial cultures based on matrix-assisted laser desorption ionization–time of flight mass spectrometry (MALDI-TOF MS), but this approach has a complex sample pretreatment process (26). In contrast, our selected RNA biomarkers could yield an accurate and sensitive response after a minimum of 5 min of antibiotic exposure, thereby establishing a rapid molecular AST method. The quantitative reference of qRT-PCR was 16S rRNA, thus eliminating the influence of cell density. Several recent reports also support the use of RNA transcripts to allow the rapid identification of methicillin-resistant S. aureus (MRSA), vancomycin-resistant *Enterococcus* (VRE), fluoroquinolone-resistant K. pneumoniae, and azithromycin-resistant Neisseria gonorrhoeae instead of traditional AST (21, 27–29). Moreover, RNA signatures have been recognized as important tools for guiding clinical practice in the diagnosis of Parkinson’s disease, cancer, and infectious diseases (30–32). Therefore, it is of great significance that our selected RNA biomarkers yield a measurable and sensitive response for enabling rapid molecular AST of *tet*(X)-positive bacteria after a short-term antibiotic exposure.

Furthermore, our study advances the understanding of the mechanisms of action of tigecycline. A previous study showed that tigecycline slowed protein translation but enhanced ribosome synthesis (33). In the present study, the global transcriptional response revealed that both transcriptions of genes of 16 ribosomal small subunits and 17 ribosomal large subunits were significantly increased in DH5α-pUC19, implying that tigecycline may act by targeting both small and large subunits of the bacterial ribosome. Similar results have also been obtained in the transcriptomic or proteomic measurement of expression changes in other species, such as Streptococcus pneumoniae and Haemophilus influenzae, after treatment with translational inhibitors, such as tetracycline or chloramphenicol (34, 35). Consistent with our findings, several studies have shown that tigecycline had a higher affinity with the 70S ribosomes than that of tetracyclines (36). Furthermore, compared with DH5α-pUC19, a considerably different global transcriptional response was observed in DH5α-pUC19-*tet*(X4), which lived normally at 2-μg/ml tigecycline by inactivating all tetracyclines. In agreement with our results, a minimal RNA transcriptomic response was also found in drug-resistant K. pneumoniae and A. baumannii after antibiotic exposure compared with drug-susceptible organisms (21, 22).

In conclusion, we successfully established a rapid and complete molecular AST assay based on significantly different transcriptome responses of *tet*(X)-negative and -positive bacteria after tigecycline exposure. Candidate RNA biomarkers were verified successfully using qRT-PCR in E. coli isolates across temporal shifts and tigecycline concentration shifts and in other variants of *tet*(X). The accuracy of this tigecycline susceptibility determination based on selected RNA biomarkers was over 87% correlated with traditional MIC and PCR results. Notably, this RBAST method has been validated and can be extended to distinguish other *tet*(X) variant-carrying pathogens. Although this research has provided a proof of principle, considerable additional work in still necessary to yield a clinic-ready, RNA-based diagnostic tool for infectious diseases.

## MATERIALS AND METHODS

### Bacterial strains.

E. coli DH5α was used as a reference strain in this study. A panel of 21 tigecycline-resistant clinical E. coli strains isolated from pork (see Table S1 in the supplemental material) was chosen and preserved in the College of Veterinary Medicine, Yangzhou University, China.

### MIC and PCR determination.

The broth microdilution method was used to determine MIC according to the Clinical and Laboratory Standards Institute (CLSI) guidelines using E. coli ATCC 25922 as the control. Briefly, 2-fold dilutions of tigecycline (Solarbio, Beijing, China) ranging from 0.25 to 128 μg/ml were dispensed in a 96-well plate. Each well was plated with 10^6^ CFU/ml of bacteria. Inoculated plates were incubated at 37°C for 18 h in the dark. Measurements were performed in triplicates, and positive and negative controls were included for each MIC determination. After incubation, MIC was determined as the lowest concentration of tigecycline with no visible bacterial growth. Tigecycline-resistant strains were defined as having MIC values of >2 μg/ml. All E. coli isolates were screened for the presence of the *tet*(X) gene through PCR using primers as previously described (6, 7). PCR products were purified and subjected to Sanger sequencing to confirm the genetic identity.

### Plasmid and strain construction.

The standard *tet*(X4) gene and other variants of *tet*(X) with their own promoters were amplified by PCR using KOD One PCR master blue mix (Toyobo, Osaka, Japan), except for *tet*(X6), *tet*(X6.2), and *tet*(X6.3), which have no function in pUC19. The primers and templates used for amplificon are shown in Table S2 in the supplemental material. Purified nucleic acid was cloned into plasmid pUC19. The constructed plasmids were then transformed into DH5α competent cells. The complete coding DNA sequences (CDSs) of *tet*(X6), *tet*(X6.2), and *tet*(X6.3) were cloned into pET23a (+) under the T7 promoter using NdeI and BamHI. Luria broth (LB) agar plates with ampicillin (100 μg/ml) and tigecycline (2 μg/ml) were used for transformant screening. Positive clones were confirmed by Sanger sequencing. E. coli DH5α carrying pUC19-*tet*(X4) was used as the engineered *tet*(X4)-mediated tigecycline-resistant strain. E. coli DH5α transformed with the blank plasmid pUC19 was considered the control for the engineered tigecycline-susceptible strain.

### Antibiotic exposure for sequencing and RNA extraction.

The engineered *tet*(X4)-mediated tigecycline-resistant strain [DH5α-pUC19-*tet*(X4)] and *tet*(X4)-negative strain (DH5α-pUC19) from a single colony were cultured in LB broth supplied with ampicillin (100 μg/ml) and were incubated overnight at 37°C to an optical density at 600 nm (OD_600_) of 1. The incubations were split into two groups, as follows: (i) the tigecycline exposure at the breakpoint concentration by CLSI (2 μg/ml) at 37°C for 60 min was the treated group, and (ii) another group without tigecycline treatment was the control. Subsequently, the supernatants were removed by centrifugation, and the samples were stored immediately in liquid nitrogen. The same procedure was repeated when qRT-PCR validation was performed. Samples of different groups were collected and extracted using the EASYspin Plus kit (Aidlab, Beijing, China) according to the manufacturer’s instructions. Triplicate RNA samples were prepared considering biological variability.

### RNA sequencing and data preprocessing.

Total RNA was extracted from the cells using the TRIzol reagent according to the manufacturer’s instructions (InvitroGen, Carlsbad, CA), and genomic DNA was removed using DNase I (Takara, Shiga, Japan). Then, the RNA quality was determined using the 2100 bioanalyzer (Agilent, Palo Alto, CA) and quantified using the ND-2000 instrument (NanoDrop, Wilmington, DE). Only high-quality RNA samples (OD_260/280_, 1.8 to 2.0; OD_260/230_, ≥2.0; RNA integrity number (RIN), ≥6.5; 28S:18S ≥1.0, ≥100 ng/μl, ≥2 μg) were used to construct the sequencing library. The transcriptome sequencing (RNA-seq) library was prepared using the TruSeq RNA sample preparation kit from Illumina (San Diego, CA) with 2 μg of total RNA. After quantification with a TBS380 instrument, the library was sequenced with the Illumina HiSeq × 10 system (2 × 150-bp read length) after being analyzed using the Illumina genome analyzer (GA) pipeline (v.1.6), for which 150-bp paired-end reads were obtained. The reads were aligned to the E. coli K-12 strain (NCBI reference sequence NC_000913.3).

### Differential expression analysis and principal-component analysis.

Differential expression analysis was performed using the edgeR, DESeq2, and DESeq packages. Principal-component analysis (PCA) was performed on the samples with the FactoMineR PCA function and plotted using ggplot2. Differentially expressed genes that enriched the Gene Ontology (GO) pathways were explored using Goatools (https://github.com/tanghaibao/GOatools) with corrected a *P* value of ≤0.05. The confidence intervals (CIs) of the accuracy of RBAST in clinical isolates were calculated using the exact binominal method provided by the UCSF online calculator (http://sample-size.net/confidence-interval-proportion/).

### Data availability.

RNA-sequencing data have been deposited in the National Center for Biotechnology Information (NCBI) Sequence Read Archive (SRA) database (PRJNA759745).
