# Supplementary material for: Rapid and Accurate Antibiotic Susceptibility Determination of tet(X)-Positive E. coli Using RNA Biomarkers
Source: Microbiol Spectr. 2021 Oct 27;9(2):e00648-21. doi: 10.1128/Spectrum.00648-21 (PMC8549723; doi:10.1128/Spectrum.00648-21)
Supplement: SUPPLEMENTAL FILE 1 — Supplemental material. Download Spectrum.00648-21-s0001.pdf, PDF file, 0.9 MB [file spectrum.00648-21-s0001.pdf]

Supplementary figures

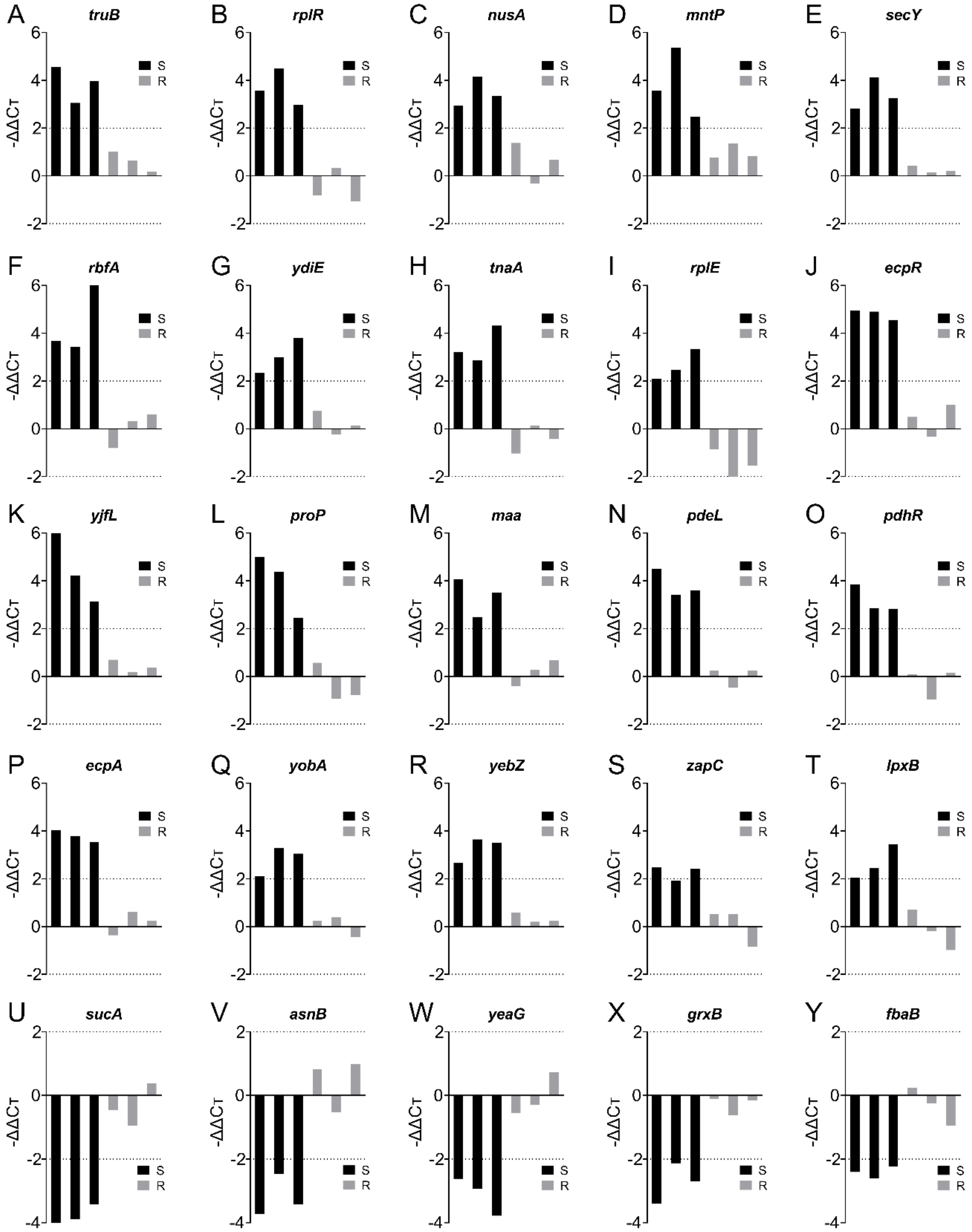

Supplementary figure 1 RBAST distinguishes *tet*(X4)-negative and -positive trains. Quantitative real-time PCR of 25 tigecycline-specific susceptible RNA biomarkers across three *tet*(X4)-negative and three *tet*(X4)-positive tigecycline-resistant *E. coli* strains after tigecycline exposure relative to their own control. Black bars indicate *tet*(X4)-negative

isolates and grey bars indicate *tet*(X4)-positive isolates. 16S rRNA was used as a reference gene.

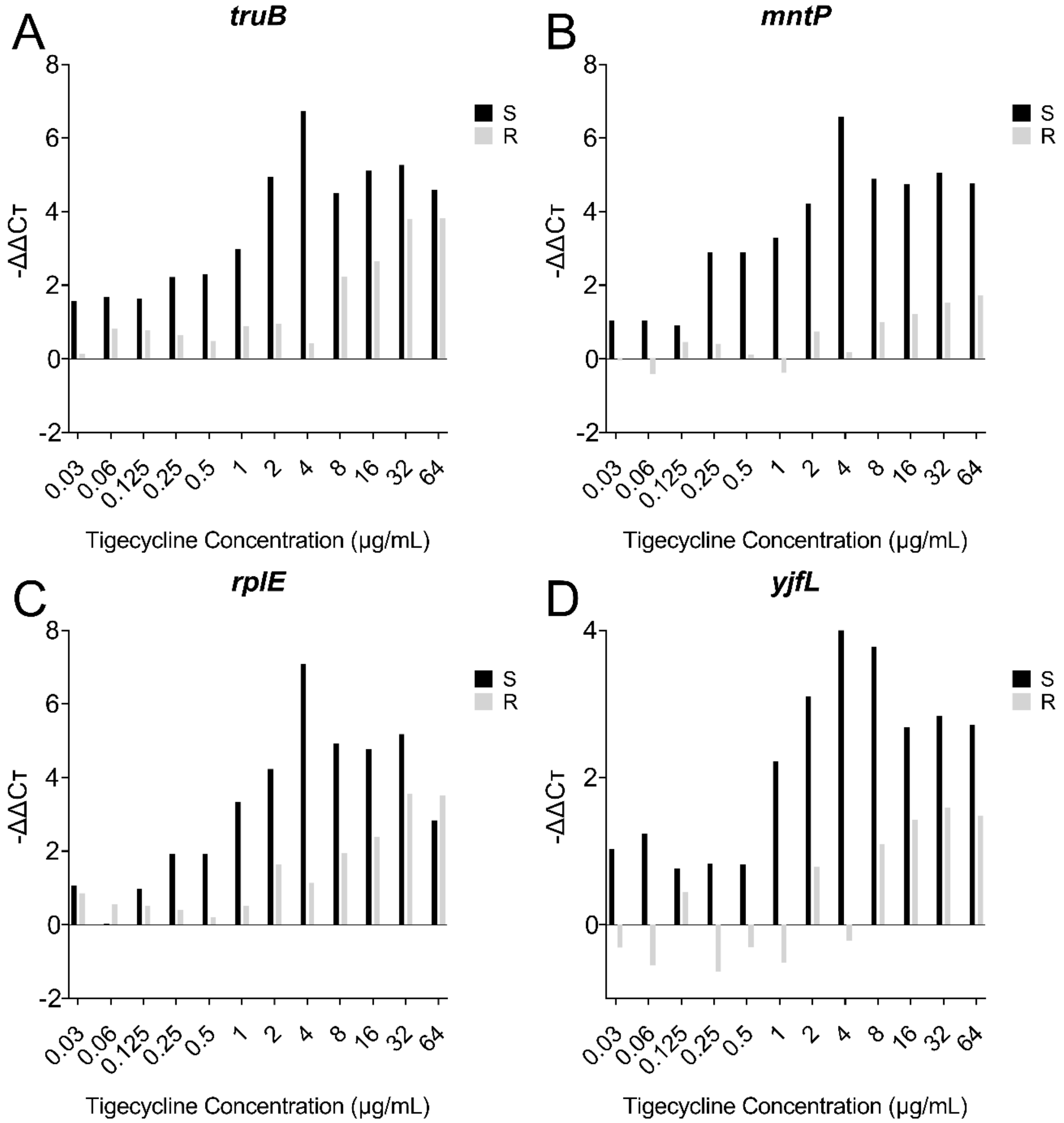

Supplementary figure 2 Expression of selected RNA biomarkers upon different tigecycline exposure concentrations.

Histogram of *truB* (**A**), *mntB* (**B**), *rplE* (**C**) and *yjfL* (**D**) biomarkers demonstrated the most sensitive susceptibility information across the MIC range of tigecycline. Black bars indicate *tet*(X4)-negative *E. coli* ATCC25922 and grey bars indicate *tet*(X4)-positive tigecycline-resistant isolate RW7-1. 16S rRNA was used as a reference gene.

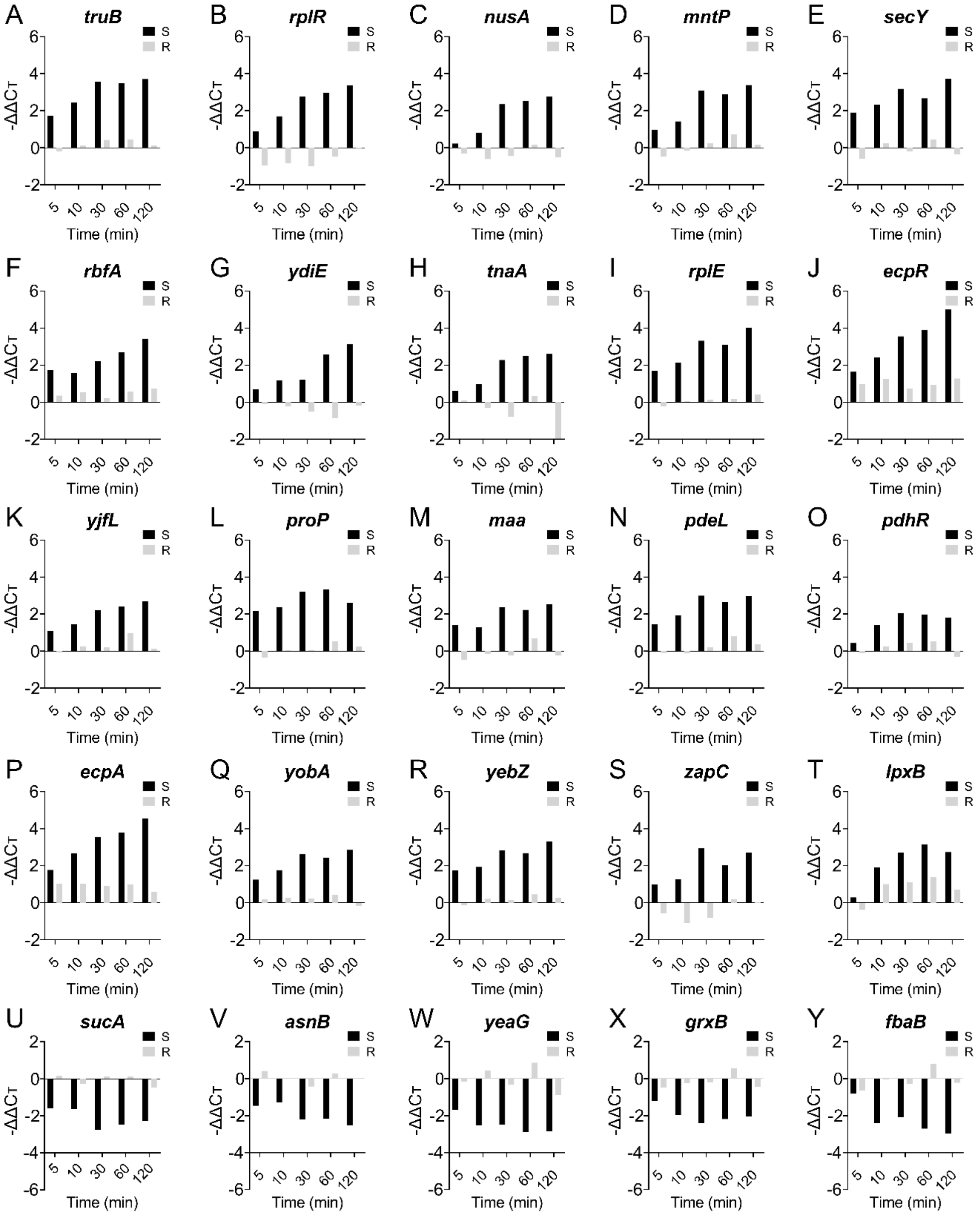

Supplementary figure 3 Expression of candidate RNA biomarkers upon different tigecycline exposure times.

Histogram of 25 differentially expressed RNA biomarkers across exposure duration of tigecycline. Black bars indicate *tet*(X4)-negative *E. coli* ATCC25922 and grey bars indicate *tet*(X4)-positive tigecycline-resistant isolate RW7-1. 16S rRNA was used as a reference gene.

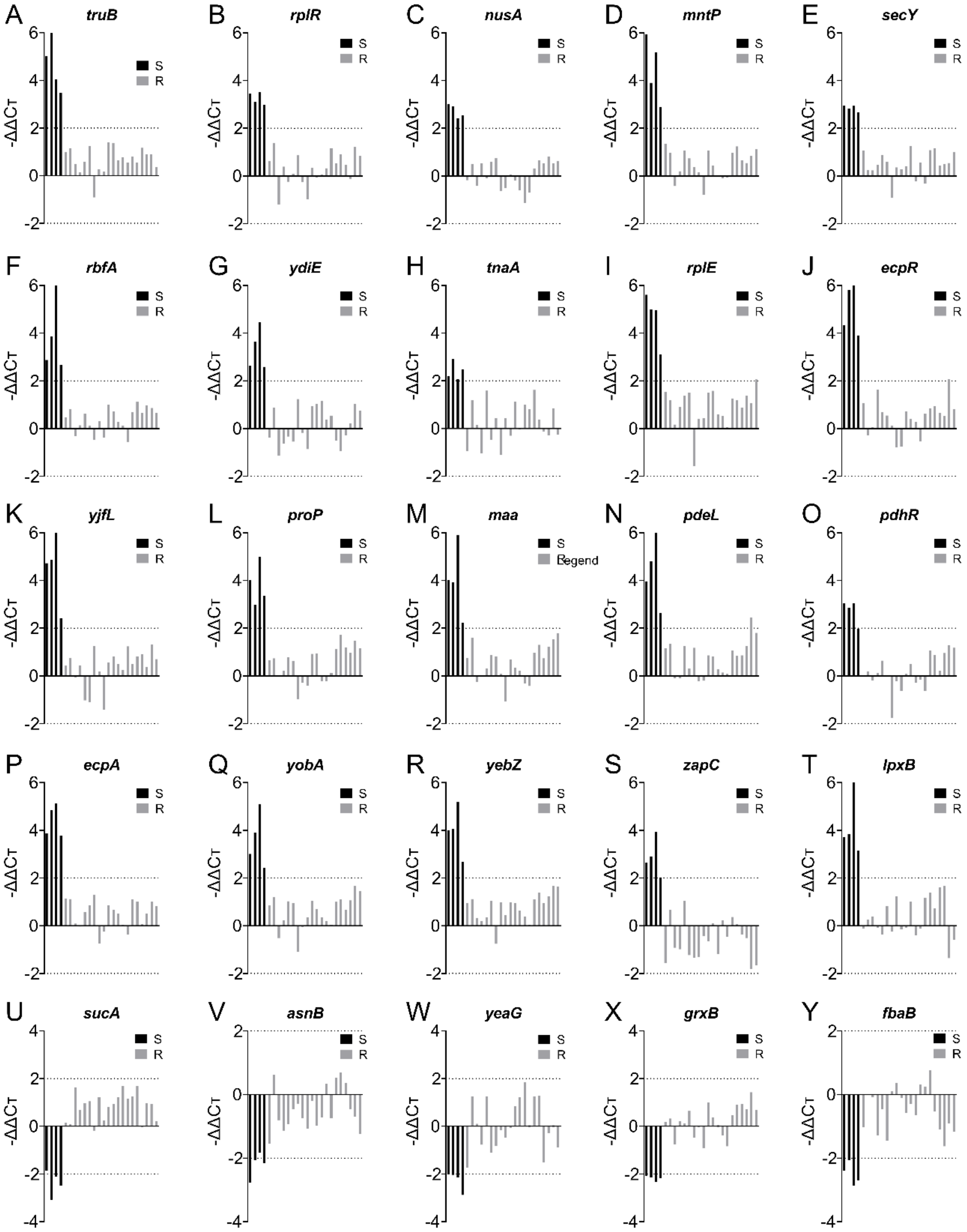

Supplementary figure 4 RBAST detects different *tet*(X) variants using the candidate RNA biomarkers.

Histogram of 25 differentially expressed RNA biomarkers validation across *tet*(X4)-negative and different variants of *tet*(X)-positive tigecycline-resistant after tigecycline exposure relative to their own control. Black bars indicate *tet*(X4)-negative strains and grey bars indicate different variants of *tet*(X)-positive tigecycline-resistant constructions. 16S rRNA was used as a reference gene.

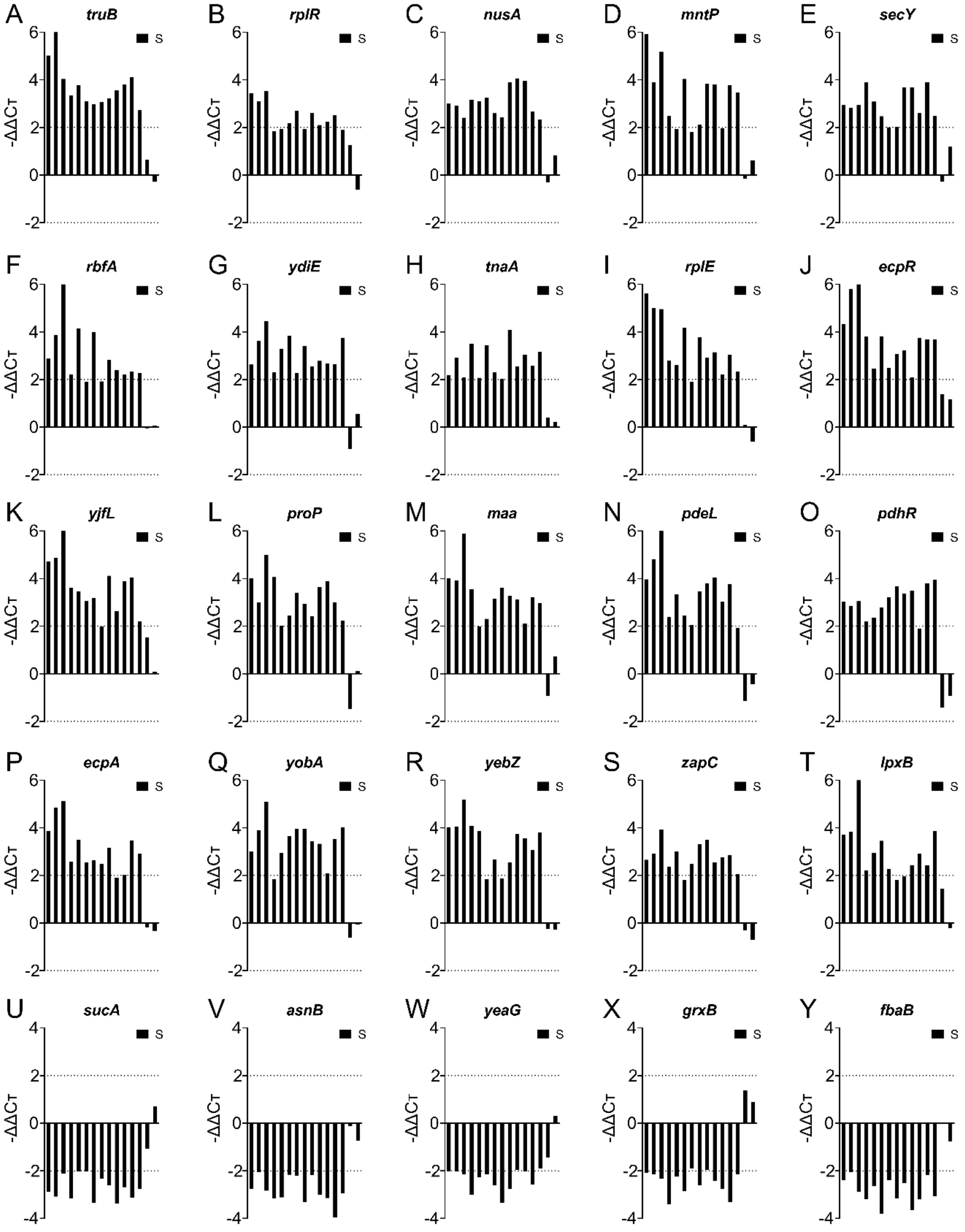

Supplementary figure 5 RBAST accurately classifies *tet*(X)-negative *E. coli* isolates. Histogram of 25 candidate RNA biomarkers across clinical *tet*(X)-negative clinical isolates after tigecycline exposure relative to their own control. 16S rRNA was used as a reference gene.

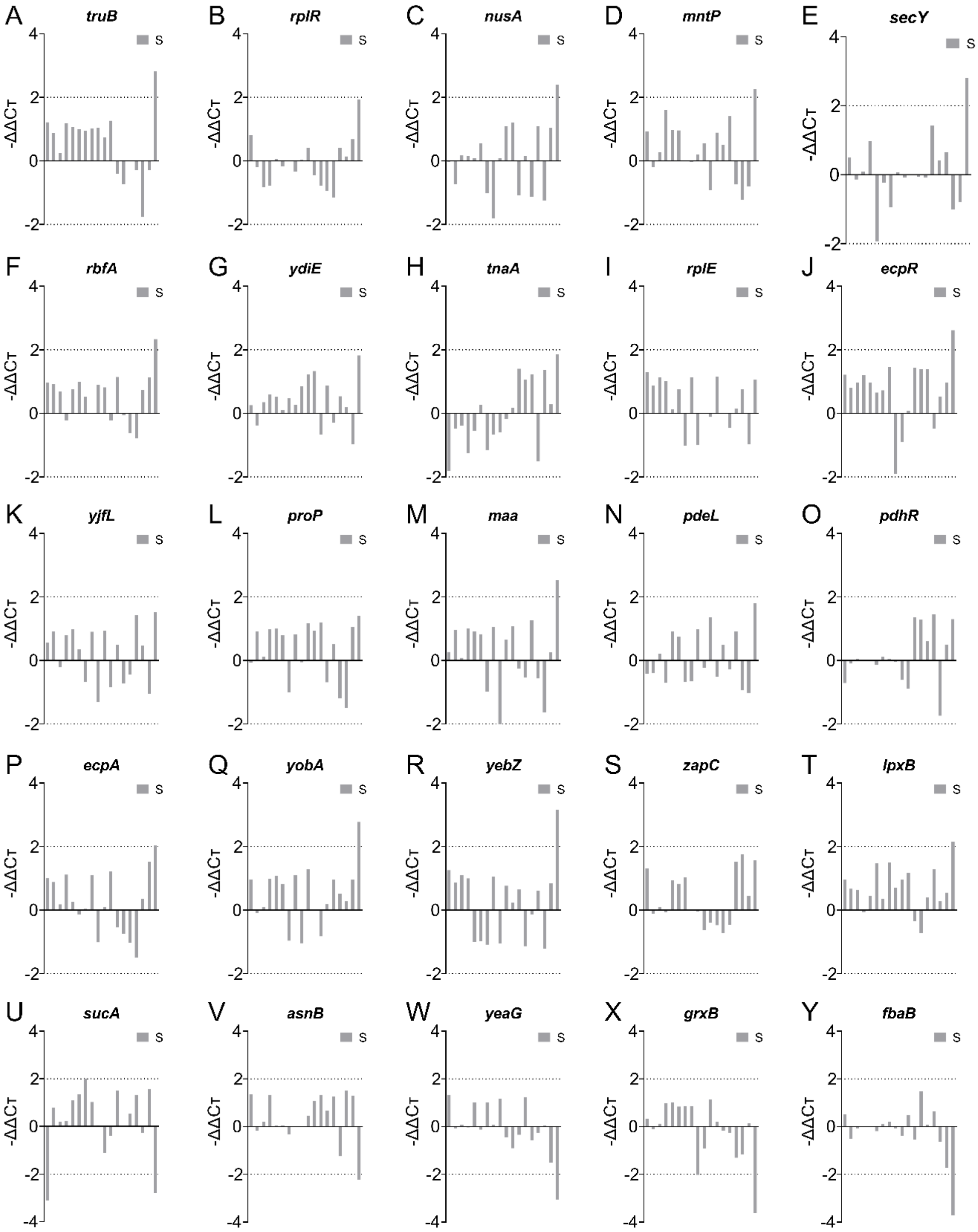

Supplementary figure 6 RBAST accurately classifies *tet*(X)-positive *E. coli* isolates. Histogram of 25 candidate RNA biomarkers across clinical *tet*(X)-positive tigecycline-resistant isolates after tigecycline exposure relative to their own control. 16S rRNA was used as a reference gene.
